# Assessment of causal relationships between omega-3 and omega-6 polyunsaturated fatty acids in autoimmune rheumatic diseases: a brief research report from a Mendelian randomization study

**DOI:** 10.3389/fnut.2024.1356207

**Published:** 2024-05-28

**Authors:** Xiao Xu, Xu Xu, Mohammad Ali Zakeri, Shu-Yun Wang, Min Yan, Yuan-Hong Wang, Li Li, Zhi-ling Sun, Rong-Yun Wang, Lin-Zhong Miao

**Affiliations:** ^1^School of Nursing, Nantong Health College of Jiangsu Province, Nantong, China; ^2^Department of Geriatrics, Renmin Hospital of Wuhan University, Wuhan, China; ^3^Non-Communicable Diseases Research Center, Rafsanjan University of Medical Sciences, Rafsanjan, Iran; ^4^Department of Postgraduate, St. Paul University Philippines, Tuggegarau, Philippines; ^5^Department of Epidemiology, School of Public Health, Changzhou University, Changzhou, China; ^6^Faculty of Health and Welfare, Satakunta University of Applied Sciences, Pori, Finland; ^7^Department of Rheumatology, The Affiliated Hospital of Nanjing University of Chinese Medicine, Nanjing, China; ^8^Department of Epidemiology, School of Public Health, Nanjing University of Chinese Medicine, Nanjing, China; ^9^Department of Rheumatology, Zhejiang Chinese Medical University, Hangzhou, China; ^10^Department of Nursing, Children’s Hospital of Soochow University, Soochow University, Suzhou, China

**Keywords:** omega-3, omega-6, polyunsaturated fatty acids, autoimmune rheumatic diseases, Mendelian randomization study

## Abstract

**Background:**

Currently, the association between the consumption of polyunsaturated fatty acids (PUFAs) and the susceptibility to autoimmune rheumatic diseases (ARDs) remains conflict and lacks substantial evidence in various clinical studies. To address this issue, we employed Mendelian randomization (MR) to establish causal links between six types of PUFAs and their connection to the risk of ARDs.

**Methods:**

We retrieved summary-level data on six types of PUFAs, and five different types of ARDs from publicly accessible GWAS statistics. Causal relationships were determined using a two-sample MR analysis, with the IVW approach serving as the primary analysis method. To ensure the reliability of our research findings, we used four complementary approaches and conducted multivariable MR analysis (MVMR). Additionally, we investigated reverse causality through a reverse MR analysis.

**Results:**

Our results indicate that a heightened genetic predisposition for elevated levels of EPA (OR_IVW_: 0.924, 95% CI: 0.666–1.283, *P*_IVW_ = 0.025) was linked to a decreased susceptibility to psoriatic arthritis (PsA). Importantly, the genetically predicted higher levels of EPA remain significantly associated with an reduced risk of PsA, even after adjusting for multiple testing using the FDR method (*P*_*IVW*–FDR–corrected_ = 0.033) and multivariable MR analysis (*P*_MV-IVW_ < 0.05), indicating that EPA may be considered as the risk-protecting PUFAs for PsA. Additionally, high levels of LA showed a positive causal relationship with a higher risk of PsA (OR_IVW_: 1.248, 95% CI: 1.013–1.538, *P*_IVW_ = 0.037). It is interesting to note, however, that the effects of these associations were weakened in our MVMR analyses, which incorporated adjustment for lipid profiles (*P*_MV-IVW_*>* 0.05) and multiple testing using the FDR method (*P*_IVW–FDR–corrected_ = 0.062). Moreover, effects of total omega-3 PUFAs, DHA, EPA, and LA on PsA, were massively driven by SNP effects in the *FADS* gene region. Furthermore, no causal association was identified between the concentrations of other circulating PUFAs and the risk of other ARDs. Further analysis revealed no significant horizontal pleiotropy and heterogeneity or reverse causality.

**Conclusion:**

Our comprehensive MR analysis indicated that EPA is a key omega-3 PUFA that may protect against PsA but not other ARDs. The FADS2 gene appears to play a central role in mediating the effects of omega-3 PUFAs on PsA risk. These findings suggest that EPA supplementation may be a promising strategy for preventing PsA onset. Further well-powered epidemiological studies and clinical trials are warranted to explore the potential mechanisms underlying the protective effects of EPA in PsA.

## 1 Introduction

Autoimmune rheumatic diseases (ARDs) comprise a collection of systemic inflammatory disorders (gout, juvenile idiopathic arthritis, ankylosing spondylitis, psoriatic arthritis, and others) characterized by autoimmune-induced damage to various organs and systems (1). The prevalence of ARDs varies among different populations, but overall, these diseases are known to be quite prevalent, particularly among women and older adults (2). ARDs not only result in significant disability but also hinder daily functioning, work performance, and productivity (3). Furthermore, the presence of comorbidities, such as cardiovascular diseases and metabolic syndrome, significantly impacts the life expectancy, overall health, and quality of life of those affected (4). The chronic pain, physical limitations, and fatigue associated with ARDs can also heavily influence mental well-being, contributing to elevated rates of psychological distress (5).

The recent American College of Rheumatology Guideline (6) has provided very low to moderate evidence regarding the importance of reducing saturated fats intake and incorporating more polyunsaturated fatty acids (PUFAs) into the diet for individuals with rheumatic conditions. PUFAs play crucial roles in regulating inflammatory response and cell signaling, modulating blood sugar and lipid metabolism, and involving antithrombotic processes, among other physiological functions (7, 8). Omega-3 PUFAs and omega-6 PUFAs are two major classes of PUFAs (9). Importantly, the quantification of omega-3 PUFAs relies on the detection of a signal shift caused by the position of the omega-3 double bond. The total concentrations of α-linolenic acid, eicosapentaenoic acid (EPA), docosahexaenoic acid (DHA), and other omega-3 PUFAs are collectively referred to as total omega-3 fatty acids. The synthesis of long-chain omega-3 PUFAs (EPA and DHA) from α-linolenic acid involves a series of elongation, desaturation, and β-oxidation processes during fatty acid metabolism. Crucially, the fatty acid desaturase (FADS) gene encodes delta-6 desaturase, which plays a pivotal role in regulating this metabolic pathway (10).

Previous observational studies have investigated the impact of omega-3 PUFA supplementation on individuals with PsA. Notably, randomized controlled trials have highlighted the benefits of high doses of EPA and DHA in enhancing clinical outcomes and quality of life, reducing PASI disease activity and inflammatory markers, and easing joint pain in PsA patients (11–13). Interestingly, dietary recommendations of the French Society for Rheumatology propose supporting weight loss in subjects who are overweight or obese, a Mediterranean-type diet and supplementation in polyunsaturated fatty acids, mainly omega-3 PUFAs (14). In contrast, some clinical investigations have shown no significant differences in disease activity improvement between PsA patients receiving PUFA supplementation and those receiving a regular diet (15, 16).

Previous observational studies with small sample sizes have limitations that could affect the credibility and interpretation of their findings. Confounding variables, including socioeconomic, environmental, lifestyle factors, and medication use, have the potential to influence both PUFAs concentration and the likelihood of developing ARDs (17). Additionally, the use of self-reported dietary questionnaires in previous observational studies may introduce recall bias and measurement error (18).

Given these limitations, Mendelian randomization (MR) has been recommended as a state-of-the-art statistical method in dietary studies for assessing the causal role of PUFAs in disease progression (19). This innovative method uses genetic variants to establish causality, similar to a randomized controlled trial, and can reduce the impact of confounding factors, reverse causation bias, and measurement error biases present in observational studies (20). Two recent MR studies (21, 22) have suggested that genetically determined increases in omega-3 PUFAs levels may contribute to the pathogenesis of RA and SLE. However, the causal relationship between PUFA levels and the risk of other ARDs remains unclear.

To bridge this knowledge gap, our study utilized MR techniques to assess the causal association between two categories of PUFAs (total omega-3 PUFAs and total omega-6 PUFAs), as well as their respective subtypes (omega-3 PUFAs: DHA and EPA; omega-6 PUFAs: LA and AA), and their relationship to the risk of ARDs (including JIA, SS, gout, AS, and PsA).

## 2 Materials and methods

### 2.1 Study design and ethics approval statement

**Figure 1:**
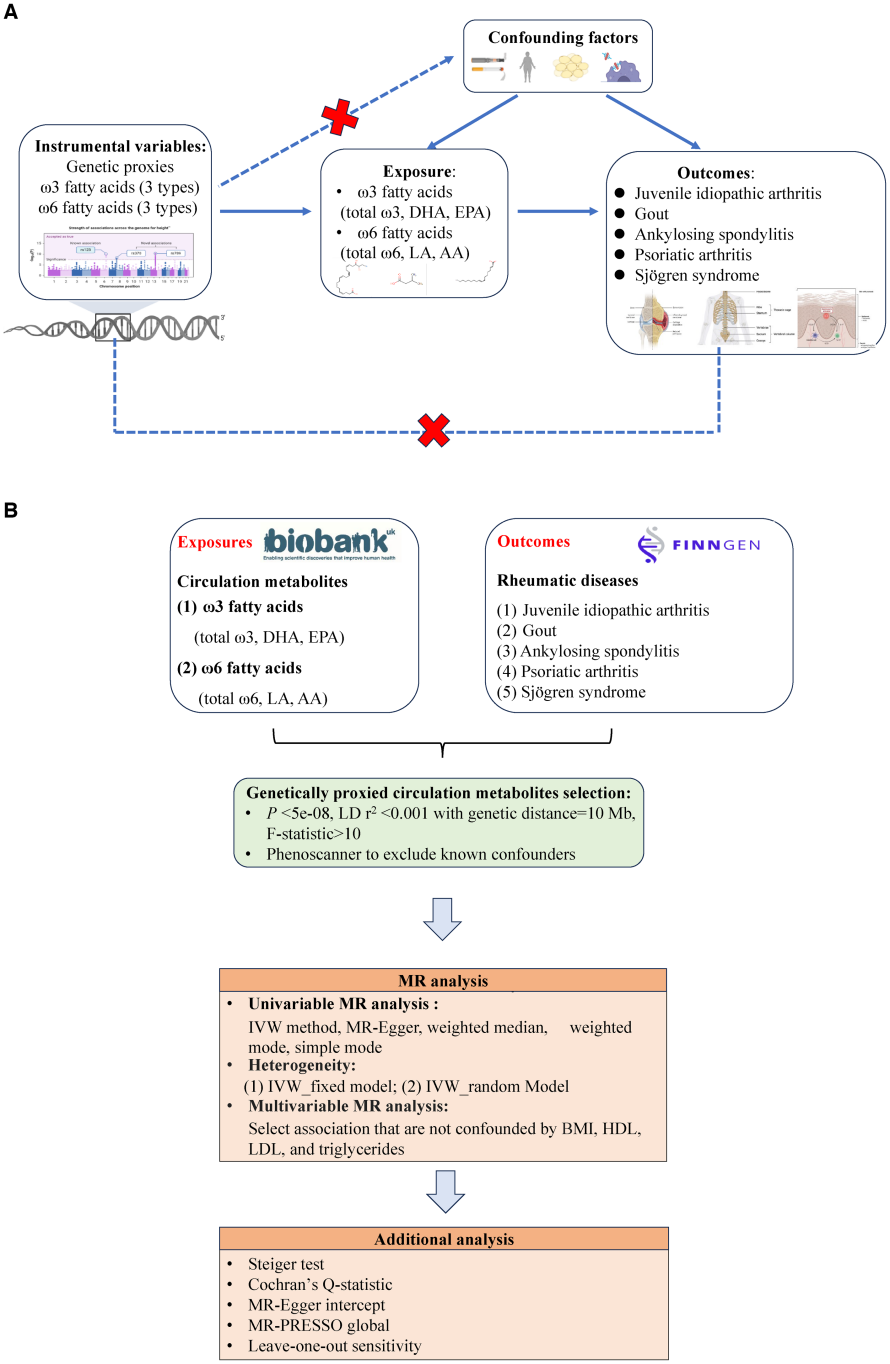
**(A)** Three key assumptions of Mendelian randomization (MR) in this study: (1) the instrumental variables (genetic proxies for omega-3 fatty acids, omega-6 fatty acids, EPA, DHA, LA, and AA) are not related to the confounders (dashed line and red 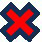); (2) the instrumental variables (genetic proxies for omega-3 fatty acids, omega-6 fatty acids, EPA, DHA, LA and AA) are related to the exposure factor (solid line), and (3) the instrumental variables (genetic proxies for omega-3 fatty acids, omega-6 fatty acids, EPA, DHA, LA, and AA) are not directly related to the outcome (rheumatic diseases: Juvenile idiopathic arthritis, Gout, Ankylosing spondylitis, Psoriatic arthritis, and Sjögren syndrome) (dashed line and red 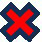). **(B)** Flowchart of overview of Mendelian randomization (MR) analysis.

Figure 1B depicts the flowchart outlining the MR analysis conducted to evaluate the causal effects of six types of PUFAs and their correlation with the risk of ARDs. The study design adhered to the MR_STROBE guidance (23) (Supplementary Table S1), and each original GWAS study involved in this MR research obtained ethical approval and informed consent from their respective institutions.

### 2.2 Data sources

Detailed information regarding the dataset used is provided in Supplementary Table S2.

#### 2.2.1 PUFAs GWAS data source

The GWAS data for the omega-3 (including DHA and EPA) and omega-6 (including LA and AA) PUFA subclasses were obtained from the Cohorts for Heart and Aging Research in Genomic Epidemiology (CHARGE) Consortium,^1^ with a sample size of 8,866 for DHA and EPA and 8,631 for LA and AA. The units for the omega-3 (DHA and EPA) and omega-6 (LA and AA) subclasses are expressed as percentages (%) of total fatty acids. Details regarding the fatty acid measurements are well described in a previous study (24). In summary, plasma phospholipids were initially isolated using thin-layer chromatography, and fatty acids such as DHA, EPA, LA, and AA were then separated using gas chromatography. The original effect estimates from the CHARGE Consortium were converted to standard units of mmol/L using GWIS (Genome-wide Inferred Study) (25), a method that approximates GWAS summary statistics (expressed as percentages of total fatty acids) for a variable that is a function of phenotypes, with available GWAS summary statistics, phenotypic means, and covariances. Detailed information can be found in Supplementary Table S2. Moreover, information on the levels of omega-3 PUFAs, omega-6 PUFAs, DHA, EPA, LA, and AA are also summarized in Supplementary Table S2.

#### 2.2.2 Outcomes data of ARDs

The largest GWAS summary data for five different ARDs were freely accessible from the Juvenile Idiopathic Arthritis Genetics Consortium (juvenile idiopathic arthritis) (26) and the FinnGen Biobank (Sjögren’s syndrome, gout, ankylosing spondylitis, and psoriatic arthritis) (27). In the Juvenile Idiopathic Arthritis Genetics Consortium, nine cohorts, including the Boston Children’s JIA Registry, British Society of Pediatric and Adolescent Rheumatology Study Group, Childhood Arthritis Prospective Study, Childhood Arthritis Response to Medication Study, German Society for Pediatric Rheumatology (GKJR), JIA gene expression study, NIAMS JIA genetic registry, TREAT study, and United Kingdom Juvenile Idiopathic Arthritis Genetics Consortium, comprised individuals of European descent from the US, UK, and Germany (26). The FinnGen Biobank study is a nationwide collection of genotyped samples from Finnish individuals containing genetic and lifelong health record data from all participants. This allows for the investigation of shared and distinct genetic landscapes associated with Sjögren’s syndrome, gout, ankylosing spondylitis, and psoriatic arthritis, providing an opportunity for GWAS and cross-disease analyses to better understand potential shared genetic contributors. Finns, as isolated populations, have a more homogeneous genetic makeup than other populations.

The Juvenile Idiopathic Arthritis Genetics Consortium dataset consists of 2,816 cases and 13,056 controls related to JIA outcomes. Furthermore, the sample sizes for the FinnGen Biobank were as follows: psoriatic arthritis (cases *N* = 8,075, controls *N* = 330,975), gout (cases *N* = 1,699, controls *N* = 21,639), ankylosing spondylitis (cases *N* = 1,462, controls *N* = 164,682), and Sjögren’s syndrome (cases *N* = 1,290, controls *N* = 214,145). Diagnoses of ARDs are primarily based on the following ICD-10 codes: juvenile idiopathic arthritis (ICD-10 M08, M09*), psoriatic arthritis (ICD-10 M07.1*, M07.1*L40.5, M07.2*, M07.2*L40.5, M07.3*, M07.3*L40.5), gout (ICD-10 M10, M10.9, M10.0), ankylosing spondylitis (ICD-10 M45), and Sjögren syndrome (M35.0). Detailed information can be found in Supplementary Table S2.

### 2.3 Selection of optimal instrumental variables

The selection of optimal IVs should comply with the following three key assumptions of Mendelian randomization (MR): (i) the IVs selected for analysis should be robustly associated with PUFAs; (ii) the IVs should be independent of potential confounders; and (iii) the IVs should only affect the outcome of ARDs through the mediation of PUFAs, indicating the absence of horizontal pleiotropy (28) (Figure 1A). To ensure the validity of the second MR assumption (exclusion-restriction), we established a strict threshold for the *p* value of outcomes (ARDs) at 5*10^−8^. Only instrumental variables (IVs) with linkage disequilibrium <0.001 after clumping within a 10,000 kb window were selected. The adjusted Cochran’s Q statistic was utilized to detect any violations of exclusion-restriction in the multivariable analysis, as indicated by a significant Q statistic (29). In addition, we rigorously tested compliance with the third assumption of MR, the independence assumption. This was done by utilizing various methods, such as PhenoScanner, to eliminate any instruments exhibiting horizontal pleiotropy (30). Furthermore, we evaluated for horizontal pleiotropy using MR–Egger regression by analyzing its intercept terms and employing the MR-PRESSO method (31).

To ensure comprehensive results, IVs for PUFAs concentrations were obtained using *p* < 5 × 10^−8^, as per previous research (32). Additionally, to minimize the impact of linkage disequilibrium (LD), a clumping process with R^2^ = 0.001 and a genetic distance of 10,000 kb was applied using the R package. Proxy SNPs with LD (R^2^ > 0.8) were used as substitutes for absent SNPs in our study (33). Subsequently, the researchers cross-referenced the Phenoscanner database to confirm that the chosen SNPs were not linked to any established confounding factors. The F-statistic [formula: (R^2^/(1-R^2^)) × ((N – K − 1)/K)] was then calculated for each exposure. SNPs with an F-statistic less than 10 were excluded to avoid potential bias from weak IVs (34). Finally, SNPs with inconsistent alleles between the exposure (PUFAs) and outcome (ARDs) samples, as well as palindromic SNPs, were discarded.

### 2.4 Statistical analysis

The IVW high-efficiency method was utilized as the primary analysis to determine the causal relationship between six PUFAs traits (total omega-3 PUFAs, DHA, EPA, total omega-6 PUFAs, LA, and AA), and the risk of ARDs, such as Sjögren’s syndrome, gout, JIA, ankylosing spondylitis, and PsA (35). Additional methods including MR-Egger, weighted median, weighted mode, and simple mode were also employed to assess the reliability of the results (36, 37).

During the confounding analysis, we examined each SNP and its corresponding proxy in the PhenoScanner GWAS and ensemble databases (38) to evaluate their relationships with various confounding traits, such as immune-related diseases, smoking, alcohol consumption, fasting blood glucose, chronic kidney disease, C-reactive protein levels, and type 2 diabetes mellitus, in relation to PUFAs and the risk of PsA. This thorough examination allowed us to identify potential pleiotropic effects (39). SNPs that displayed associations with these confounders at a significance level of *p* < 1 × 10^−5^ were excluded from the analysis, and the instrumental variable weighting (IVW) method was then reapplied to ensure the elimination of horizontal pleiotropy and validate the robustness of our Mendelian randomization results.

In this research, the chosen SNPs related to total omega-3, DHA, EPA, total omega-6, LA, and AA were located within or in close proximity to genes involved in lipid transport and metabolism in a broader sense (Supplementary Files 3–8). Consequently, traits associated with lipid metabolism, such as BMI, HDL, LDL, and triglycerides, are considered potential confounders for the risk of ARDs (40). BMI data were obtained by analyzing results from the largest meta-analysis of genome-wide association studies (GWASs) for BMI, which included summary statistics from the Genetic Investigation of Anthropometric Traits (GIANT) consortium (41) combined with GWAS data on BMI from UK Biobank participants of European descent, totaling approximately 700,000 individual (42). Genome-wide association data for blood lipid levels, including HDL, LDL, and triglyceride levels, were obtained from the UK Biobank; these data included information on more than 13.7 million single nucleotide polymorphisms (SNPs) and were obtained from the Neale laboratory (43). Model adjustments in this UK Biobank GWAS from the Neale laboratory accounted for age, age^2, sex (inferred from genotype), interaction terms for age*sex and age^2*sex, as well as the first 20 principal components. All measured serum biomarkers exhibited an approximately normal distribution, albeit with a positive skew. To maintain consistency, inverse rank-normalized data were utilized for all biomarkers. Multivariable MR (MVMR) analysis, an extension of univariate MR (UVMR), was employed to isolate the direct causal effects of the exposure on the outcome, considering the pleiotropic factor as a covariate in the regression model (44). In this analysis, four lipid metabolism-related traits—BMI, HDL, LDL, and triglycerides—were included as covariates using MVMR to mitigate potential biases from confounding variables. The criteria for selecting instrumental variables (IVs) required that the IVs be associated with one of the exposures while remaining unassociated with potential confounders or the outcome. The thresholds for the *p* value and R^2^ remained consistent with those mentioned for UVMR. Subsequently, after excluding duplicate SNPs, proxies were identified for SNPs not present in other exposures using the ‘TwoSampleMR’ R package. Moreover, palindromic or ambiguous SNPs were eliminated. Finally, SNPs linked to either the outcome or the confounders were also removed to address potential horizontal pleiotropy. For MVMR analysis, the MVMR-IVW method was primarily utilized. Furthermore, the MR-Pleiotropy Residual Sum and Outlier methods (MR-PRESSO) were employed to detect overall horizontal pleiotropy (global test), identify specific outliers (outlier test), and recalculate effect estimates following outlier removal in MVMR analysis, ensuring robust estimation of causal effects even in the presence of weak instruments or pleiotropy.

To establish a more thorough examination of the causal relationship between PUFAs and PsA, we conducted multiple tests (involving various PUFAs, such as omega3/omega6, and their subtypes) with Benjamini–Hochberg FDR correction. The significance of the associations between multiple PUFAs and PsA was determined when the FDR-corrected *p* value decreased to less than 0.05. Associations with *p* values less than 0.05 but FDR-corrected *p* values greater than 0.05 were considered suggestive of associations between PUFAs and PsA, following the approach outlined by Story and Tibshirani (45). Furthermore, we assessed the statistical power (above 80%) of potential PUFAs that could be considered candidates at a significance level of 0.05 utilizing an mRnd power calculator tool available online at https://sb452.shinyapps.io/power based on the methodology outlined by Burgess et al. (46).

Furthermore, to mitigate the bias from reverse causation, a reverse causality analysis and Steiger filtering analysis were also conducted in this study. The IVW method was employed as the main analytical approach in the reverse Mendelian randomization (MR), aimed at assessing the causal connection between the susceptibility to ARDs and polyunsaturated fatty acids (PUFAs) characteristics. The Cochran’s Q test and MR-Egger intercept were utilized for the evaluation of heterogeneity and horizontal pleiotropy of the IVs (47). Additionally, the MR-PRESSO method was applied to identify and address potential pleiotropy (37). Additionally, a leave-one-out sensitivity analysis was performed, in which the effect of the six PUFAs traits on the results was recalculated after removing each IV individually (37).

All statistical analyses were performed using the ‘TwoSampleMR’, ‘MVMR’, ‘MRPRESSO’, and ‘MendelianRandomization’ packages (R version 4.1.2).

## 3 Results

Following the established criteria for selecting instrumental variables (IVs), including the clustering and standardization of single nucleotide polymorphisms (SNPs), elimination of palindromic SNPs, and exclusion of SNPs with an *F* value below 10, we identified 10–41 SNPs strongly correlated with total omega-3 PUFAs, 4–12 SNPs strongly associated with DHA, 3–17 SNPs strongly linked to EPA, 16–55 SNPs strongly correlated with total omega-6 PUFAs, 7–18 SNPs strongly associated with LA, and 1–8 SNPs strongly correlated with AA for various ARDs. The F-statistics for different types of circulating PUFAs ranged from 490.35 to 12.25 (Supplementary Tables S3–S8).

Through single-locus Mendelian randomization analysis, only the most significant SNPs from chromosomes 11 or 6 were selected to predict PUFA levels, with F-statistic values ranging from 22.56 to 168.99. Specifically, rs174564, rs174546, and rs174538 in the fatty acid desaturase (FADS) gene cluster served as genetic instruments for total omega-3 PUFAs, DHA, and EPA, respectively. The genetic instruments for LA and AA were rs99780 and rs472031 in FADS2 and FADS3, respectively. Finally, rs3734854 in C6orf11 functions as a genetic instrument for total omega-6 PUFAs.

### 3.1 Causal effects of total omega-3 PUFAs, DHA, and EPA on ARDs

**Figure 2:**
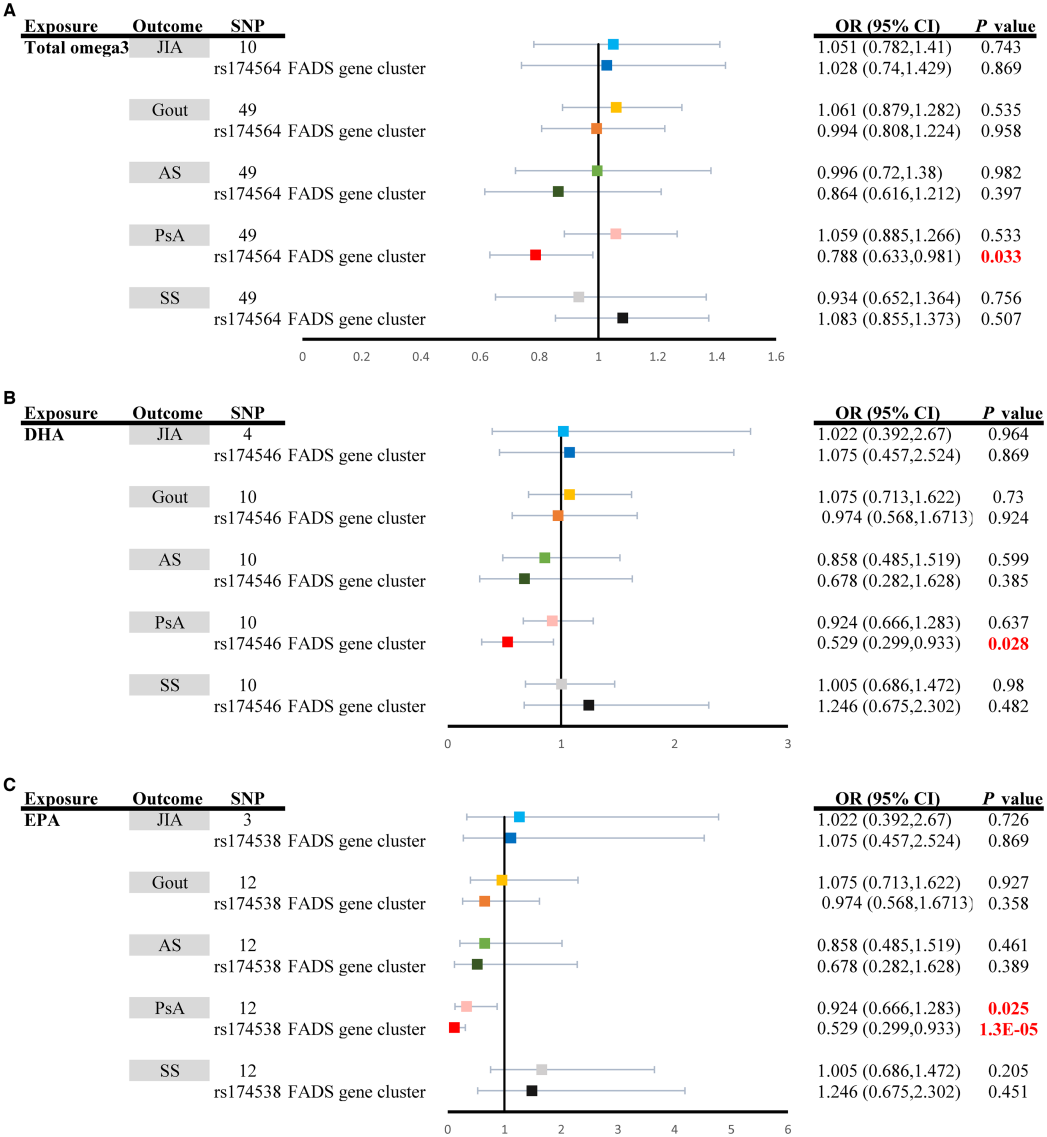
Univariable causal effects of **(A)** total omega3 polyunsaturated fatty acids, **(B)** DHA, and **(C)** EPA on investigated outcomes (JIA: light shades of blue, Gout: light shades of orange, AS: light shades of green, PsA: pink and SS: grey); Univariable causal effects of **(A)** total omega3 polyunsaturated fatty acids, **(B)** DHA, and **(C)** EPA on investigated outcomes (JIA: blue, Gout: orange, AS: green, PsA: red and SS: black) via the *FADS* gene cluster. DHA, docosahexaenoic acid; EPA, eicosapentaenoic acid; FADS, fatty acid desaturase; JIA, Juvenile idiopathic arthritis; AS, Ankylosing spondylitis; SS, Sjögren’s syndrome; PsA, Psoriatic arthritis.

Figure 2 shows the effects of omega-3 PUFAs, including total omega-3 PUFAs, DHA, and EPA, on ARD risk. Considering total omega-3 fatty acids as a whole, little evidence has indicated its protective effect on ARD risk [JIA: (OR_IVW_: 1.0151, 95% CI: 0.782–1.410, *P*_IVW_ = 0.743); Gout: (OR_IVW_: 1.061, 95% CI: 0.879–1.282, *P*_IVW_ = 0.535); ankylosing spondylitis (OR_IVW_: 0.996, 95% CI: 0.720–1.380, *P*_IVW_ = 0.982), PsA (OR_IVW-random_: 1.059, 95% CI: 0.885–1.266, *P*_IVW_ = 0.533), and Sjögren’s syndrome (OR_IVW_: 0.934, 95% CI: 0.652–1.364, *P*_IVW_ = 0.756)] (Supplementary Table S9; Figure 2A). Moreover, higher concentrations of DHA had a potential effect on decreasing the risk of ARDs, although the evidence was weaker due to the wide confidence interval [JIA: (OR_IVW_: 1.022, 95% CI: 0.392–2.670, *P*_IVW_ = 0.964); Gout: (OR_IVW_: 1.075, 95% CI: 0.713–1.622, *P*_IVW_ = 0.730); ankylosing spondylitis (OR_IVW_: 0.858, 95% CI: 0.485–1.519, *P*_IVW_ = 0.599); PsA (OR_IVW-random_: 0.924, 95% CI: 0.666–1.283, *P*_IVW_ = 0.637); and Sjögren’s syndrome (OR_IVW_: 1.005, 95% CI: 0.686–1.472, *P*_IVW_ = 0.980)] (Supplementary Table S10; Figure 2B). In contrast, genetically increased levels of EPA had a causal effect on the decreased risk of PsA (OR_IVW_: 0.924, 95% CI: 0.666–1.283, *P*_IVW_ = 0.025) (Supplementary Table S11; Figure 2C). Nevertheless, there was no evidence of a causal link between the levels of EPA in the bloodstream and the risk of other ARDs (*p* > 0.05) (Supplementary Table S11; Figure 2C). The four complementary methods produced results consistent with those of the IVW method (Supplementary Tables S9–S11).

### 3.2 Causal effects of total omega-6 PUFAs, LA and AA on ARDs

**Figure 3:**
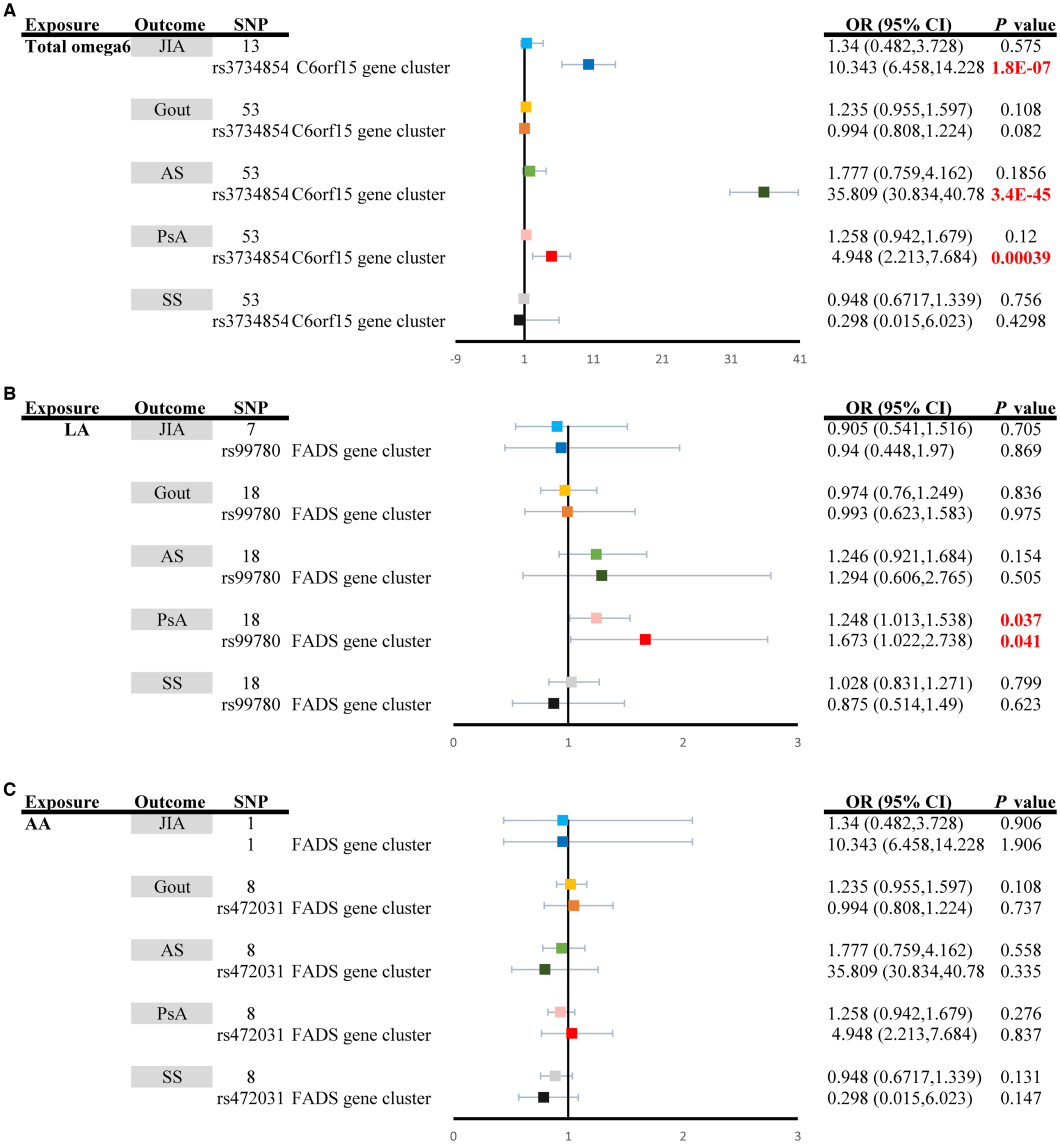
Univariable causal effects of **(A)** total omega6 polyunsaturated fatty acids, **(B)** LA, and **(C)** AA on investigated outcomes (JIA: light shades of blue, Gout: light shades of orange, AS: light shades of green, PsA: pink and SS: grey). Univariable causal effects of **(A)** total omega6 polyunsaturated fatty acids, **(B)** LA, and **(C)** AA on investigated outcomes (JIA: blue, Gout: orange, AS: green, PsA: red and SS: black) via the *FADS* gene cluster and the *C6orf15* gene cluster. LA, Linoleic acid; AA, Arachidonic acid; FADS, fatty acid desaturase; JIA, Juvenile idiopathic arthritis; AS, Ankylosing spondylitis; SS, Sjögren’s syndrome; PsA, Psoriatic arthritis.

The random effect IVW analysis revealed that genetically determined total omega-6 PUFA levels were not associated with the risk of developing any type of ARD, as demonstrated by the results for JIA (OR_IVW_: 1.340, 95% CI: 0.482–3.728, *P*_IVW_ = 0.5751), Gout (OR_IVW_: 1.235, 95% CI: 0.955–1.597, *P*_IVW_ = 0.108), Ankylosing spondylitis (OR_IVW_: 1.777, 95% CI: 0.759–4.162, *P*_IVW_ = 0.1856), PsA (OR_IVW_: 1.258, 95% CI: 0.942–1.679, *P*_IVW_ = 0.120), and Sjögren’s syndrome (OR_IVW_: 0.948, 95% CI: 0.6717–1.339, *P*_IVW_ = 0.756) (Supplementary Table S12; Figure 3A). Moreover, circulating AA was not significantly associated with an increased risk of JIA (OR_IVW_: 1.340, 95% CI: 0.482–3.728, *P*_IVW_ = 0.5751), gout (OR_IVW_: 1.235, 95% CI: 0.955–1.597, *P*_IVW_ = 0.108), ankylosing spondylitis (OR_IVW_: 1.777, 95% CI: 0.759–4.162, *P*_IVW_ = 0.558), PsA (OR_IVW_: 1.258, 95% CI: 0.942–1.679, *P*_IVW_ = 0.276), or Sjögren’s syndrome (OR_IVW_: 0.948, 95% CI: 0.6717–1.339, *P*_IVW_ = 0.131). However, genetically increased levels of LA had a causal effect on the increased risk of PsA (OR_IVW_: 1.248, 95% CI: 1.013–1.538, *P*_IVW_ = 0.037) (Supplementary Table S13; Figure 3B). When employing the fixed-effect mode of the IVW method, no genetic association was identified between circulating LA and other types of ARDs, including Sjögren’s syndrome, JIA, ankylosing spondylitis, and PsA (*p* > 0.05) (Supplementary Table S14; Figure 3C). The additional methods yielded results consistent with those of the primary IVW method (Supplementary Tables S12–S14).

### 3.3 Sensitivity analysis in the FADS gene region

The MR estimates of total omega-3 PUFAs, DHA, EPA, and LA in PsA patients were strongly affected by SNPs in the *FADS* gene region (rs174564 for total omega-3 fatty acid, rs174546 for DHA, rs174538 for EPA, rs99780 for LA, rs472031 for AA, and rs3734854 for total omega-6 PUFAs) (Figures 2, 3). As shown in Figure 2, the MR results of the single FADS SNPs revealed causal effects of total omega-3 fatty acids, DHA, and EPA on the risk of PsA (*P*_IVW_ < 0.05). In contrast, single-locus MR analysis revealed that the C6of15 gene cluster of total omega-6 PUFAs strongly affected the risk of developing JIA, AS, and PsA (*P*_IVW_ < 0.05). Moreover, a single FADS SNP in LA had a positive effect on increasing PsA risk (OR, 1.673; 95% CI, 1.022–2.738) but had less of an effect on other types of ARD (Figure 3).

### 3.4 Confounding analysis

The possible traits of two types of PUFAs, EPA and LA (*P*_IVW_ < 0.05), are summarized in Figures 2, 3. There were three potentially pleiotropic SNPs (rs7480288 in EPA and rs1260326 and rs629301 in LA) identified by PhenoScanner (Table 1; Supplementary Table S15), and after removing these SNPs, the results were still consistent with previously reported significant associations [EPA: OR_IVW_: 0.438, 95% CI: 0.199–0.965, *P*_IVW_ = 0.040; LA: OR_IVW_: 1.297, 95% CI: 1.051–1.601, *P*_IVW_ = 0.0155] (Table 2).

**Table 1:** SNPs associated with confounders.

PUFAs	SNP	Trait	*P* value	Sources	References
EPA	rs7480288	Core binding factor acute myeloid leukemia	3.00 E-14	NHGRI-EBI GWAS catalog	PMID:27903959
LA	rs1260326	Alcohol consumption; Fasting blood glucose; Type 2 diabetes; Chronic kidney disease; C-reactive protein levels	1.00 E-21	NHGRI-EBI GWAS catalog	PMID:28937693 PMID:31217584 PMID:29632382 PMID:20383146 PMID:36376304
	rs629301	C-reactive protein levels	6.00 E-132	NHGRI-EBI GWAS catalog	PMID:27286809

EPA, eicosapentaenoic acid; LA, α-linolenic acid.

**Table 2:** The association between genetic proxies for PUFA traits and PsA after excluding pleiotropic SNPs.

PUFAs	Outcome	Method	OR (95%CI)	*P* value
EPA	PsA	IVW	0.438 (0.199, 0.965)	0.040
LA	PsA	IVW	1.2967 (1.0506, 1.6005)	0.0155

IVW, inverse-variance weighted; PsA, psoriatic arthritis; EPA, eicosapentaenoic acid; LA, α-linolenic acid.

### 3.5 Sensitivity analysis

The outcomes from Cochran’s Q-statistic, which included the IVW test and MR–Egger regression method, showed no significant heterogeneity among these selected SNPs (*p* > 0.05) (Table 3). The MR-PRESSO test demonstrated no evidence of horizontal pleiotropy or any obvious outliers for the IVs used in this MR study (*p* > 0.05). MR–Egger regression intercept analysis yielded similar findings (*p* > 0.05), suggesting no significant directional horizontal pleiotropy (Table 3).

**Table 3:** Heterogeneity, horizontal pleiotropy test, and steiger filtering.

Exposure	Outcome	Heterogeneity			Pleiotropy		MRPRESSO Global test	Steiger filtering (Steiger_pval)
		Method	Q value	Q_pval	Intercept	*P* value	*P* value	
EPA	Psoriatic arthritis	MR Egger	15.144	0.127	0.127	0.142	0.112	TRUE (*p* = 1.748577E-207)
		Inverse variance weighted	18.987	0.061				
LA	Psoriatic arthritis	MR Egger	18.125	0.317	0.002	0.911	0.307	TRUE (*p* = 4.549E-210)
		Inverse variance weighted	19.286	0.3123				

Moreover, our leave-one-out analysis indicated that the MR results were primarily driven by SNPs in the FADS gene cluster (rs174538 in EPA and rs99780 in LA); hence, desaturation steps during polyunsaturated fatty acid biosynthesis might play a key role in this relationship (Supplementary Figure S1). Finally, to mitigate the potential for reverse causality between circulating EPA and LA and the risk of PsA, the MR-Steiger test was utilized to confirm the direction of all significant MR estimates of causality between genetically determined two subtypes of omega-3 PUFAs (EPA and LA) and PsA (Table 3). Furthermore, the statistical power of potentially eligible candidate PUFAs after multiple adjustments was consistently greater than 80% (Supplementary Table 15).

### 3.6 Multivariate MR analysis

**Figure 4:**
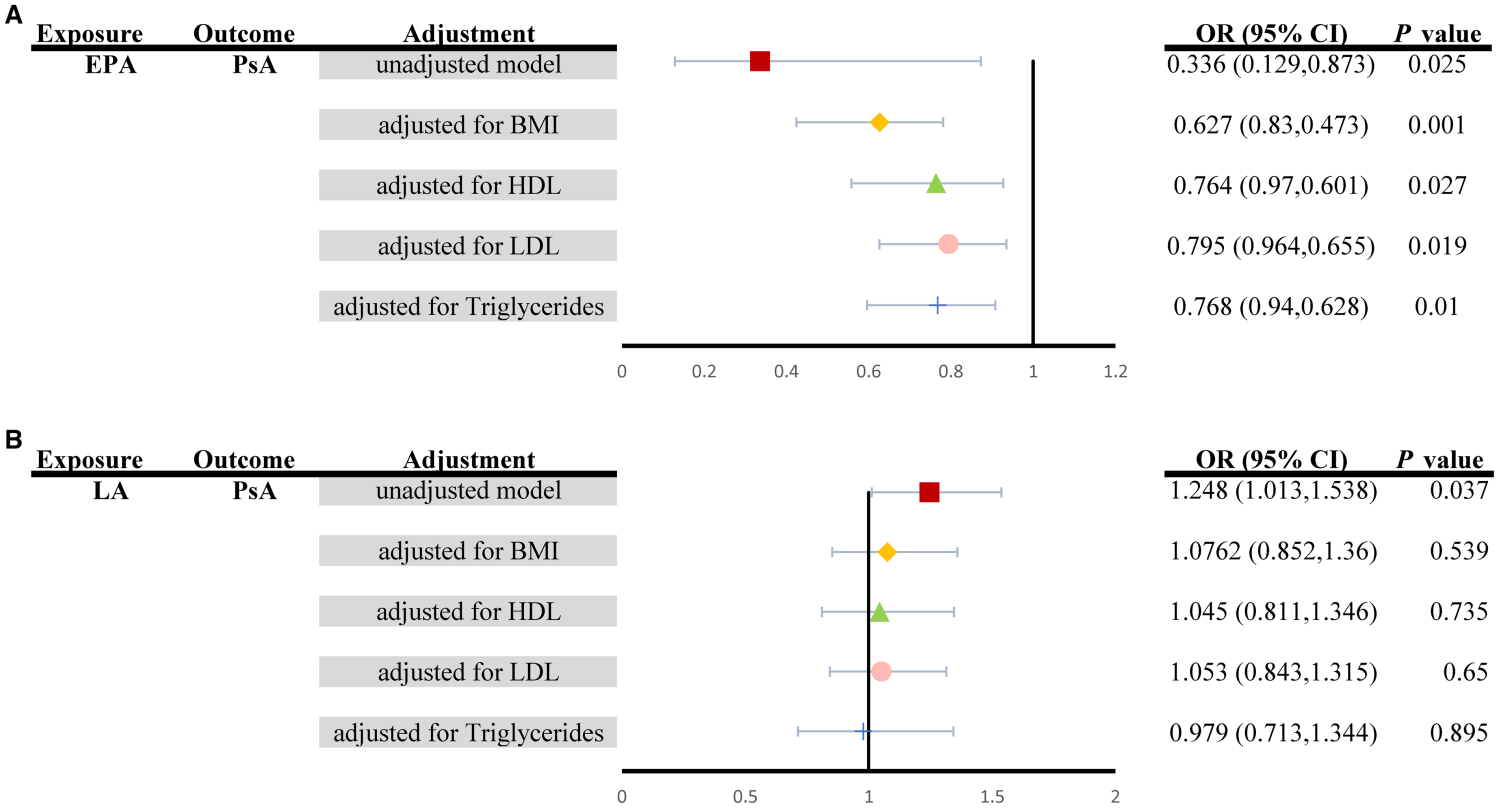
Summary of MVMR causal effects for **(A)** EPA and **(B)** LA levels on the risk of PsA when controlling for four potential confounders (Unadjusted model: red square; BMI: yellow diamond; HDL: green triangle; LDL: pink circle; triglycerides: blue cross). EPA, Eicosapentaenoic acid; LA, α-linolenic acid; PsA, Psoriatic Arthritis; MVMR, Multivariable MR.

The results of the multivariate MR analysis are shown in Figure 4. since univariate MR (UVMR) showed no causal relationship between four types of PUFAs (total omega-3, DHA, total omega-6, AA) and the risk of four types of ARDs (JIA, gout, AS, and SS); thus, for multivariate MR analysis, we explored only the causal relationship between two types of PUFAs (LA and EPA) and the risk of PsA.

The associations of genetically predicted levels of EPA with the risk of PsA were similar in multivariable MR and univariable MR (Figure 2). More specifically, in multivariable MR analysis controlling for BMI, HDL, LDL and triglycerides individually, there was strong evidence for a negative causal effect of EPA on the risk of PsA (unadjusted model: OR*_MV-IVW_*: 0.336, 95% CI: 0.129–0.873, *P_MV-IVW_* = 0.025; controlling for BMI: OR*_MV-IVW_*: 0.627, 95% CI: 0.830–0.473, *P_MV-IVW_* = 0.001; controlling for HDL: OR*_MV-IVW_*: 0.764, 95% CI: 0.970–0.601, *P_MV-IVW_* = 0.027; controlling for LDL: OR*_MV-IVW_*: 0.795, 95% CI: 0.964–0.655, *P_MV-IVW_* = 0.019; controlling for triglycerides: OR*_MV-IVW_*: 0.768, 95% CI: 0.940–0.628, *P_MV-IVW_* = 0.010), although the effect size of the Furthermore, the MVMR_PRESSO results indicated no evidence of horizontal pleiotropy in this MVMR analysis (MR-PRESSO Global Test RSSobs = 341.171, MR-PRESSO Global Test *p* value = 0.266). However, it should be noted that other associations may have been influenced by potential confounding variables to some extent (Figure 4A). Specifically, for genetically predicted LA, a positive association with PsA observed using univariable MR (unadjusted model: OR*_MV-IVW_*: 1.248, 95% CI: 1.013–1.538; *P_MV-IVW_* = 0.037) was not detected via multivariable MR analysis (controlling for BMI: OR*_MV-IVW_*: 1.076, 95% CI: 0.852–1.360; *P_MV-IVW_* = 0.539; controlling for HDL: OR*_MV-IVW_*: 1.045, 95% CI: 0.811–1.346; *P_MV-IVW_* = 0.735; controlling for LDL: OR*_MV-IVW_*: 1.053, 95% CI: 0.843–1.315; *P_MV-IVW_* = 0.650; controlling for triglycerides: OR*_MV-IVW_*: 0.979, 95% CI: 0.713–1.344; *P_MV-IVW_* = 0.895) (Figure 4B).

### 3.7 Benjamini-Hochberg FDR correction

For the total omega-3 PUFAs and its subtype dataset, the results from the FDR correction revealed that higher relative abundances of EPA retained significant causal relationships with a lower risk of PsA (*P*_IVW–FDR–corrected_ = 0.033). For the total omega-6 PUFAs and its subtype dataset, after applying this multiple-testing correction, there were suggestive associations but not significant associations between the levels of LA and PsA (*P*_IVW–FDR–corrected_ = 0.062).

### 3.8 Reverse causality

All SNPs from the reverse MR analysis are presented in Supplementary Table 16. Notably, IVW analysis indicated no reverse causality between EPA/LA and the risk of PsA (*P_IVW_* > 0.05), which suggested the absence of reverse causality (Table 4). PsA may not be the cause of these specific PUFAs but rather the outcome of them. Additionally, there was no evidence of heterogeneity or horizontal pleiotropy in the causal relationships between PsA and EPA/LA (*p* > 0.05) (Table 4). The results of the MR Steiger test further support the causal direction from the EPA/LA to the PsA, rather than the reverse (Table 4).

**Table 4:** Inverse variance weighted (IVW)-based MR model estimates of the causal relationships between the risk of psoriatic arthritis and candidate EPA/LA and tests for heterogeneity and horizontal pleiotropy.

Exposure	Outcome	Heterogeneity			Pleiotropy		MRPRESSO Global test	Steiger filtering (Steiger_pval)
		Method	Q value	Q_pval	Intercept	*P* value	*P* value	
Psoriatic arthritis	EPA	MR Egger	4.400	0.623	0.005	0.534	0.207	TRUE (*p* = 4.06E-25)
		Inverse variance weighted	4.835	0.680				
Psoriatic arthritis	LA	MR Egger	8.132	0.521	−0.009	0.112	0.134	TRUE (*p* = 6.41E-62)
		Inverse variance weighted	8.145	0.615				

## 4 Discussion

Our research employed MR to evaluate the causal relationship between two types of PUFAs (total omega-3 PUFAs and total omega-6 PUFAs), as well as their different subtypes (omega-3 PUFAs: DHA and EPA; omega-6 PUFAs: LA and AA), and their connection to the risk of ARDs (including JIA, SS, gout, AS, and PsA). We provided evidence supporting that increased levels of EPA are causally associated with a lower risk of PsA, but the effect on other types of ARDs, including JIA, SS, gout and AS, is relatively weaker (Figure 2). Importantly, these correlations were found to be causal in nature, even after adjusting for multiple testing using the FDR method (*P*_IVW–FDR–corrected_ = 0.033) and multivariable MR analysis (*P*_MV-IVW_ < 0.05) (Figure 4). Furthermore, we did not observe any substantial evidence of a bidirectional effect of PsA on EPA. However, we found limited evidence to support the effects of total omega-3 and DHA on the risk of ARDs. Additionally, LA was the subtype of total omega-6 PUFAs that showed significant causal estimates for a greater risk of PsA (Figure 3). It is interesting to note, however, that the effects of these associations were weakened in our MVMR analyses, which incorporated adjustment for lipid profiles (*P*_MV-IVW_ > 0.05) and multiple testing using the FDR method (*P*_IVW–FDR–corrected_ = 0.062). Moreover, the effects of total omega-3 PUFAs, DHA, EPA, and LA on PsA were massively driven by SNP effects in the *FADS* gene region (Figures 2, 3). Therefore, desaturation steps during omega-3 PUFA biosynthesis might play a critical role in the relationship between omega-3 PUFAs and the risk of ARDs. Collectively, our results suggest that supplementation with EPA (rather than total omega-3 PUFAs, total omega-6 PUFAs, DHA, LA or AA) might be a more effective strategy to prevent the onset of ARDs, especially PsA, rather than JIA, SS, gout, or AS.

Moreover, omega-3 PUFAs have been demonstrated to be associated with numerous health benefits, such as improvements in cardiometabolic risk factors, type 2 diabetes mellitus, low back pain, bipolar disorder and others (48–51). However, our MR results only suggest a causal association between genetically increased EPA levels, the total omega-6 PUFA subtype, and a decreased risk of PsA in individuals of European ancestry. Previously, several observational studies have investigated the impact of omega-3 PUFA supplementation on ARDs. Nonetheless, conflicting reports have emerged regarding whether omega-3 PUFAs actually have an effect on ARDs. For example, previous randomized controlled trials reported the potential benefits of daily omega-3 supplementation or high doses of EPA and DHA on improving clinical manifestations and health-related quality of life, reducing PASI disease activity and inflammatory markers, and alleviating joint pain in patients with PsA (11–13). However, there are also some controversies about the efficacy of omega-3 PUFA supplementation on PsA, where although omega-3 PUFA supplementation significantly reduced weight loss, waist circumference, and body fat, no differences in disease activity improvement in PsA were observed between the long-chain omega-3 PUFA supplementation group and the diet placebo group (15, 16).

However, there was no significant association between PUFAs and the risk of other ARDs. According to observational studies, low intake of omega-3 PUFAs increases the risk of radiographic axial spondyloarthritis and disease activity in AS patients in Northern and Western European populations (52, 53), and high-dose (4.55 g) omega-3 PUFA supplementation has been shown to improve functional capacity and decrease disease activity and drug consumption in AS patients in northern Sweden according to a clinical trial performed by B Sundström et al. (54). Moreover, a retrospective study (including 49 children with JIA and 29 healthy subjects aged between 3 and 18 years) from Anna Górska et al. (55) showed that omega-3 PUFAs could play a critical role in the inflammatory process and the pathogenesis of JIA in children, and omega-3 PUFA deficiency may be a risk factor for JIA; specifically, serum levels of alpha-linolenic acid (one of the main forms of omega-3 PUFAs) are significantly lower in JIA children than in healthy controls. In a double-blind randomized controlled trial, the consumption of omega-3 PUFA supplements was demonstrated to be effective as an add-on therapy to conventional nonsteroidal anti-inflammatory drug (NSAID) treatment to reduce inflammatory cytokines, including interleukin-1 (IL-1) and tumor necrosis factor-α (TNF-α), and improve the pediatric ACR response in patients with JIA (56). Additionally, a cross-sectional study performed by Carlos Y Castrejón-Morales (57) detected the intake and serum levels of omega-3 PUFAs among Mexican patients with primary Sjögren’s syndrome, and the results showed that omega-3 PUFAs may be a risk factor for Sjögren’s syndrome, which further results in chronic inflammation, including elevated CCL2 levels, in the saliva of Sjögren’s syndrome patients. Furthermore, Zhang et al. (58) examined the relationship between self-reported omega-3 PUFA supplementation and the risk of recurrent gout flares using conditional logistic regression, adjusting for various confounders, and the results showed that dietary omega-3 PUFA-rich fish consumption, when adjusted for total purine intake, was associated with a decreased risk of recurrent gout flares.

Conversely, the results of a previous cross-sectional study (59) showed that neither dietary intake of long-chain omega-3 fatty acids nor a Western diet correlated with disease activity, as assessed by the Bath Ankylosing Spondylitis Disease Activity Index (BASDAI), in AS patients. Moreover, one epidemiological study (60) from Poland showed that the association between omega-3 fatty acid and omega-6 fatty acid intake did not differ between JIA children and healthy controls. Furthermore, in a prospective, randomized, double-masked trial, omega-3 PUFA supplementation (TheraTears Nutrition®) was not found to be significantly better than conventional therapies in stimulating saliva production in American patients with Sjögren’s syndrome (61). A Cochrane systematic review (62) further evaluated the effects of omega-3 and omega-6 PUFA supplements on dry eye symptoms, and the results illustrated that the evidence of the potential benefit of long-chain omega-3/omega-6 supplementation as an adjunct in managing dry eye disease is still uncertain and inconsistent. A recent 24-week, randomized, open-label clinical trial performed by Lisa Stamp et al. (63) revealed that patients who were diagnosed with gout in Australia and New Zealand and who received 6.2 g of omega-3 fish oil daily did not have significantly lower serum urate levels than did gout patients in control groups.

The inconsistent findings from previous observational studies may be due to limited sample sizes, varied doses, different types of omega-3 PUFA supplementation, potential recall bias in dietary reporting, and inadequate intervention and follow-up periods, leading to insufficient statistical power for drawing definitive conclusions. Additionally, the diverse effects of specific omega-3 PUFA types make it challenging to conduct clinical trials that isolate the causal effect of total omega-3 PUFAs on the risk of ARDs without strict dietary modifications, resulting in inconclusive outcomes from previous research (64). This study aimed to explore the causal effects of various omega-3 PUFA types on the risk of PsA using Mendelian randomization (MR) analysis. MR analysis is a powerful tool for determining causal relationships between modifiable exposures and complex diseases. It has gained popularity in the medical literature for uncovering causal effects of different exposures on complex diseases that may not be easily discerned through traditional randomized clinical trials. Genetic information, being determined prior to confounding factors or outcomes, is minimally influenced by other clinical variables, providing more reliable results. The results of our MR study suggest that different omega-3 PUFAs may not uniformly reduce the risk of PsA, with EPA possibly being the most effective in preventing its onset. These findings could help inform future trials by emphasizing specific omega-3 PUFA types as potential intervention targets.

Previously, two comprehensive systematic reviews were conducted to examine the potential benefits of omega-3 fatty acids in the treatment of inflammatory rheumatic diseases and systemic lupus erythematosus. The findings of these reviews suggested that omega-3 fatty acids could offer therapeutic advantages for individuals suffering from these conditions. However, it is important to acknowledge the limitations identified within these reviews.

The study by Sigaux et al. (65), for example, highlighted that the sample size of patients with inflammatory rheumatic diseases included in the meta-analysis was relatively small. Most of the studies analyzed involved fewer than 30 patients who were administered polyunsaturated fatty acids (PUFAs), which may not be sufficient to establish robust conclusions regarding the positive effects of PUFAs on inflammatory rheumatic diseases. Additionally, subgroup analyses based on the Jadad score for quality did not yield significant differences. The meta-analysis also included studies that varied significantly in terms of control groups (placebo, active comparator, no comparator) and supplementation protocols (dosage, type), which contributed to the observed heterogeneity in the analysis. Notably, all randomized controlled trials (RCTs) included in the reviews reported only overall intake of omega-3 PUFAs, without specific dietary modifications focusing on individual types of omega-3 PUFAs such as eicosapentaenoic acid (EPA) or docosahexaenoic acid (DHA).

In the research conducted by Duarte-García et al. (66), the duration of the randomized controlled trials (RCTs) and the relatively small sample sizes (the largest study in the systematic review included fewer than 50 patients per group) potentially restricted the scope of the findings. The study with the longest duration included in the meta-analysis spanned 26 weeks, which limits the availability of long-term data and limits its overall applicability. Additionally, the quality of the RCTs included in the systematic review was generally subpar, undermining the credibility of the results. Furthermore, the various ethical backgrounds from which the RCTs originated may introduce bias into the systematic review. Despite this possibility, the authors did not conduct subgroup analysis to determine the heterogeneity in their study, which could impact the validity of their conclusions.

Several limitations cannot be neglected in the above two systematic reviews; thus, despite the known anti-inflammatory properties of total omega-3 PUFAs, attributed to their ability to reduce the production of cytokines (67) and C-reactive protein (CRP) (68) in previous clinical observational studies, the available data from two recent MR studies provided less convincing evidence to support the use of omega-3 PUFAs in the prevention or treatment of rheumatoid arthritis (21) and systemic lupus erythematosus (22).

Zhu et al. (21) conducted a Mendelian randomization investigation to explore the potential causal impacts of omega-3 PUFAs on rheumatoid arthritis. The outcomes of the MR analysis indicated a significant connection between genetically predicted omega-3 PUFAs and an elevated risk of RA. Notably, a detailed single SNP bias analysis identified a specific SNP, rs174564, within the FADS gene cluster among the 32 omega-3 PUFA SNPs, which exhibited a contrary effect on the previously observed positive relationship between omega-3 PUFAs and RA risk. Additionally, Wang et al. (22) investigated the connection between genetically predicted circulating omega-3 PUFAs and susceptibility to systemic lupus erythematosus (SLE). The MR analysis outcomes demonstrated a causal link between genetically determined increases in circulating omega-3 levels per standard deviation and a heightened risk of developing SLE. Conversely, there was no observed causal relationship between genetic predisposition to omega-6 fatty acid supplementation and SLE. These findings collectively indicate that genetically determined elevation in omega-3 fatty acid levels may serve as a contributing factor to the pathogenesis of SLE. In their MR study, Wang et al. (22) underscored that previous positive reports on the benefits of omega-3 PUFAs in clinical observational studies and systematic reviews could be influenced by factors such as limited study population size, variations in omega-3 supplementation doses, and follow-up durations, potentially leading to inadequate statistical power to definitively establish associations between total omega-3 PUFAs and SLE. Moreover, Wang et al. (22) emphasized the consideration of lifetime effects of SNPs in MR studies, as opposed to focusing on short-term impacts, which might elucidate the discrepancies in outcomes and potential biases observed between MR analysis and earlier clinical investigations.

Interestingly, the results from our MR study were somewhat consistent with previous MR studies related to rheumatic diseases (RA and SLE), which also indicated a positive but not significant association between genetically elevated levels of total omega-3 PUFAs and susceptibility to PsA.

The inconsistent results between previous clinical observational studies and recent MR studies may be attributed to several biological mechanisms. First, omega-3 PUFAs had been proven to regulate the balance between Th1 and Th2 (69–71). Recent mendelian randomized trials (21, 22) also showed a connection between Th2 production and ARDs. Furthermore, the use of a machine learning algorithm by Braanker et al. (72) and Elia et al. (73) revealed increased levels of Th2 cells in patients with PsA. IL-13-expressing Th2 cells were also found to be associated with the risk of developing PsA through genome-wide association (74). Therefore, based on these studies, it is suggested that increased levels of omega-3 PUFAs may potentially trigger PsA by activating Th2 cells. Second, recent Mendelian Randomization (MR) studies have shown that a specific top SNP is closely related to RA and SLE (21, 22). In this MR study, it was found that rs58542926 is highly correlated with omega-3 PUFAs (Supplementary Table S3). The rs58542926, located in the TM6SF2 gene locus, has been identified as a proxy for NAFLD. Earlier observational studies have proved an increased likelihood of NAFLD in PsA patients, with a possible connection being attributed to chronic inflammation process (75). Third, determining whether the risk of PsA is influenced more by elevated levels of short-chain PUFAs or reduced levels of long chains is a challenging task. Variations at any stage of the biosynthesis pathway can impact both short-chain and long-chain PUFAs. Since the production of omega-3 and omega-6 PUFAs shares common enzymes, it is difficult to distinguish between independent effects and potential confounding factors. For instance, a modification in the function of the elongase enzyme can alter the concentrations of both long-chain omega-3 and omega-6 PUFAs, even if the change originates from only one of these fatty acid categories.

Another plausible explanation for these different findings is that total omega-3 PUFAs comprise various fatty acids with different carbon chain lengths, bond saturation, and diverse biochemical mechanisms (76). This complexity may explain why the overall effect of total omga-3 PUFAs is diminished or challenging to decipher in relation to PsA. Hence, the specific roles and effects of individual omega-3 PUFAs, such as EPA and DHA, need to be explored more comprehensively to understand their potential benefits in PsA management. However, by implementing MR analysis, it is possible to explore the causal effects of different omega-3 PUFA types, including EPA and DHA, on the risk of complex diseases, which can fill the gaps in previous clinical trials. Thus, our current MR study suggested that supplementation with EPA rather than total omega-3 PUFAs or DHA might be a more effective strategy for preventing the onset of PsA (Figure 2).

An additional possible explanation for these varying results is that total omega-3 polyunsaturated fatty acids (PUFAs) consist of a mixture of fatty acids with different lengths of carbon chains, levels of saturation, and distinct biochemical functions. This intricate composition may result in an overall impact of total omega-3 PUFAs that is either diminished or difficult to interpret in relation to psoriatic arthritis (PsA). As a result, there is a need to delve more deeply into the specific roles and effects of individual omega-3 PUFAs, such as eicosapentaenoic acid (EPA) and docosahexaenoic acid (DHA), to grasp their potential benefits in managing PsA. Using Mendelian randomization (MR) analysis, it becomes feasible to investigate the causal effects of various types of omega-3 PUFAs, including EPA and DHA, on the risk of complex diseases. This approach can help bridge the gaps left by previous clinical trials. Consequently, our present MR study suggested that supplementation with EPA, as opposed to total omega-3 PUFAs or DHA alone, could be a more effective strategy for preventing the development of PsA (Figure 2).

Additionally, MR studies offer several advantages over traditional observational studies, including reduced bias from confounding and reverse causation and the ability to establish causality in nonrandomized settings. Additionally, effects identified using MR may also reflect maternal genetic influences that impact intrauterine exposure to fatty acids during fetal development, which could have long-lasting effects on health outcomes (77). This highlights the complex interplay between genetic factors and environmental exposures in the development of omega-3 PUFAs. Therefore, the findings from this MR study on the impacts of PUFAs on PsA should be understood as the potential risks associated with continual exposure to omega-3 PUFAs throughout one’s lifetime, or at minimum, prolonged exposure that encompasses critical periods during the early development of PsA.

Furthermore, the effects of omega-3 PUFAs on the risk of PsA were strongly influenced by SNP effects in the *FADS* gene region. Therefore, desaturation steps during omega-3 PUFA biosynthesis might play a critical role in the relationship between omega-3 PUFAs and the risk of PsA.

Previous MR studies have revealed that specific SNPs have a negative effect on the positive association between omega-3 PUFAs and the risk of rheumatoid arthritis (RA), atopic dermatitis (AD), and inflammatory bowel disease (IBD) by regulating FADS expression. According to Zhu et al.’s (22) study, rs174564, which is located in the lipid metabolic gene fatty acid desaturase 2 (FADS2) locus, is strongly associated with plasma omega-3 PUFA levels and has a negative effect on the positive association between omega-3 PUFAs and the risk of RA. Moreover, in Lin et al.’s (78) study, MR leave-one-out sensitivity analyses demonstrated that the IVW results were significantly influenced by a specific SNP (rs174546) in the analyses of omega-3 PUFAs. Estimates resulting from the Wald ratio method for single-SNP analyses revealed that rs174546 is likely to play a crucial negative role in the causal link between omega-3 PUFA levels and atopic dermatitis. Furthermore, In Jia et al.’s (10) MR study, the MR estimates of omega-3 PUFAs in inflammatory bowel disease (IBD) patients were mainly driven by SNP effects in the FADS gene region. The MR results of the single FADS2 SNP showed the causal effects of total omega-3 PUFAs, EPA, and DHA on the risk of IBD.

The paralogous cluster of FADS genes underwent evolutionary processes through gene duplication events and is located on the long arm of human chromosome 11 at the cytogenetic position 11q12–13.1. The current evidence points to FADS variation as a crucial ‘control point’ in the synthesis and levels of various biologically significant lipids and signaling molecules rich in PUFAs, many of which have implications for health and the outcomes of human diseases.

Specifically, FADS gene polymorphisms are known to be related to the amount of fatty acids in human tissues, which may modify how fatty acid-related rheumatic diseases present symptoms (79). To further explore this issue, clear differences in *FADS2* haplotypes across ancestries have been proposed to explain variable results in clinical trials involving omega-3 PUFA supplements, and dietary shifts may feasibly contribute to population differences in the rates of decrease in PsA incidence. For example, recent research findings from the VITAL trial underscore the critical importance of taking into account genetic ancestry or race, as well as FADS genotypes, when conducting omega-3 PUFA supplementation trials and making dietary recommendations. The impact of FADS variation on outcomes can often be overlooked, resulting in missed opportunities to identify subgroups that could benefit. The data of numerous African Americans who could benefit from omega-3 PUFA supplementation may have been overlooked due to the generalized reporting of negative outcomes (80). A study by Kim et al. (81) revealed that specific FADS-associated SNPs, such as rs174575 and rs2727270, were linked to insulin resistance in Korean men with type 2 diabetes. Conversely, Brenner et al. (82) reported that only rs174456 was associated with insulin resistance in individuals of European ancestry. Discrepancies in allele frequencies, environmental factors, or the presence of unique SNPs between different ethnic populations may explain these varying associations with FADS SNPs. Furthermore, Cormier et al. (83) highlighted the complexity of the relationship between omega-3 PUFA intake and type 2 diabetes mellitus (T2DM) when considering FADS polymorphisms. Omega-3 PUFA supplementation may offer protective benefits against T2DM in individuals carrying the minor allele of the FADS gene. Similarly, research by Yao et al. (84) demonstrated that Chinese individuals carrying the minor allele of the FADS SNP (rs174616) had a reduced risk of developing T2DM. Interestingly, the protective effects of dietary omega-3 PUFAs against T2DM appear to be more prominent in Asian populations than in European or North American populations. Cultural variations in food preparation methods rich in omega-3 PUFAs, as well as genetic factors such as FADS polymorphisms, likely play a significant role in influencing this disparity.

Hence, the initial findings from the research provided some understanding as to why there was no notable causal link between total omega-3 PUFAs and PsA in this particular study. However, the rs174564 SNP in the FADS gene cluster, which is specifically related to total omega-3 PUFAs, exhibited a significant inverse relationship with PsA. It is crucial to conduct further experiments to assess the impact of various types of omega-3 PUFAs on a range of immune cell subsets within the specific context of tissue residency in individuals with PsA. Moreover, taking into account specific genotypes at FADS could pave the way for customized supplementation and dietary advice for individuals with ARDs.

Eicosapentaenoic acid (EPA) and docosahexaenoic acid (DHA) are the primary constituents of long-chain omega-3 fatty acids, which are produced from α-linolenic acid through a sequence of elongation, desaturation, and β-oxidation processes. The potential benefits of EPA and DHA have been extensively studied both individually and in combination through observational research and clinical trials involving omega-3 supplementation. However, the specific impacts of EPA and DHA on the risk of psoriatic arthritis (PsA) have not been thoroughly explored. Notably, our Mendelian randomization (MR) study conducted distinct analyses and revealed compelling evidence suggesting that higher levels of EPA may be linked to a reduced likelihood of developing PsA.

Interestingly, our MR investigations imply that EPA could exert a more pronounced influence than DHA on the risk of PsA. EPA, an essential component of omega-3 polyunsaturated fatty acids (PUFAs), has been demonstrated to regulate inflammatory responses in ARDs by inhibiting the production of proinflammatory cytokines such as tumor necrosis factor-alpha (TNF-α) and interleukin-6 (IL-6), dampening the activation of immune cells such as T cells and macrophages, and mitigating inflammation and joint damage associated with arthritis (85).

Although direct comparative studies on the effects of EPA versus DHA on PsA risk remain scarce, existing research has offered insights that align with the outcomes of our MR analysis. For instance, in a study involving twenty-one asthmatic adults, EPA exhibited a greater ability than DHA to reduce the production of IL-1β and TNF-α by alveolar macrophages (86). Additionally, the Cardiovascular Health Study indicated that higher levels of plasma phospholipid EPA, rather than DHA, were associated with decreased concentrations of C-reactive protein (CRP) (87). Considering these findings, EPA may hold greater relevance in the context of preventing PsA.

## 5 Limitations and strengths

Several limitations need to be acknowledged in our study. Firstly, it is important to note that this study on MR only encompassed individuals of European descent. Secondly, we were unable to conduct the nonlinear MR study to explore the associations between PUFAs and ARDs due to the lack of individual-level data. Thirdly, significant heterogeneity was observed in some of our results, in line with previous dietary-related MR studies (51, 88). Nevertheless, the use of IVW random model effectively controlled pooled heterogeneity and mitigated its impact on the study (88). Lastly, it is worth noting that serum metabolic levels of PUFAs can be influenced by various environmental factors such as gender, ethnicity, disease activity, and the use of immunosuppressive agents. Unfortunately, the original studies did not provide enough detailed demographic data, making it unfeasible to perform additional subgroup analysis until now. Lastly, the impact of gender differences on MR estimates is a crucial aspect that should be addressed in the limitations section. Gender stratification is known to significantly enhance the credibility and reliability of MR studies by allowing for the examination of potential effect modifications based on sex. Variations in genetic associations between males and females can arise due to hormonal profiles, physiological responses, and susceptibility to diseases. Incorporating gender differences in analysis can lead to the identification of sex-specific genetic determinants influencing disease risk and guide personalized medicine strategies (89).

The UK Biobank^2^ is a comprehensive prospective cohort study that has acquired a vast array of genetic, phenotypic, and clinical data from more than 500,000 individuals in the United Kingdom. The extensive genome-wide genotyping data available in the UK Biobank serve as a valuable resource for conducting MR studies, particularly in investigating the causal relationships between risk factors and complex diseases. Through gender stratification within the UK Biobank dataset, researchers can gain valuable insights into the sex-specific genetic landscape of disease risk and potential targets for therapeutic interventions.

Currently, our research team has submitted a request for access to the UKB database, which is currently undergoing the review process. Consequently, we were unable to perform sex stratification analysis in the present MR study. However, in future investigations, following approval from the UK Biobank, we intend to utilize gender stratification analysis to explore the impact of various PUFAs on ARDs based on UK Biobank data.

Notably, the data used in our MR study were derived from distinct patient populations obtained from the UK Biobank (exposures) and the FinnGen biobank (outcomes), effectively preventing sample overlap. Sample overlap poses a potential limitation in Mendelian randomization studies, where the use of the same genetic variants in multiple analyses can result in correlated estimates and potential biases in causal inference (90). This underlines the rationale for not conducting gender stratification analysis using UK Biobank data for outcomes in our study. The detrimental effects of sample overlap have been extensively discussed in prior MR research, including the introduction of bias and the inflation of causal estimates’ precision. When identical individuals are utilized for both exposure and outcome analyses, the instrumental variable assumptions of MR may be compromised, leading to distorted causal effect estimates.

In studies focusing on the impact of PUFA interventions through MR analysis, a common issue arises due to sample overlap. Specifically, when exposed to the UK Biobank, researchers frequently opt to complement this with outcome data from the FinnGen Biobank. For example, Dai et al. (32) investigate whether a causal relationship exists between the concentration of circulating PUFAs and chronic pain as well as the direction of this association. In the GWAS data section, GWAS data on circulating PUFA concentrations (exposures) were obtained from the UK Biobank and included 114,999 European participants. The GWAS data for pain in eight body parts were obtained from the Finland database (outcomes).

It is essential to acknowledge that the FinnGen biobank does not offer individual-level data on gender, which could hinder the execution of gender-specific analyses in Mendelian randomization studies. It is worth mentioning that a number of MR studies conducted using the ARDs as the outcome in the FinnGen biobank also refrained from gender-specific analyses due to the absence of individual-level gender data in the biobank. For example, Tang et al. (91) explored the causal effect of the gut microbiota on spondyloarthritis and its subtypes. GWAS summary data for the different subtypes of SpA (AS, PsA, and EA) were obtained from the FinnGen Biobank. Patients with AS, PsA, and EA were selected using ICD-10 diagnostic codes. However, due to the lack of individual-level data on gender in the FinnGen biobank, Tang et al. did not perform gender-specific analyses in this MR study.

Interestingly, in previous traditional observational studies, when the researchers stratified the analysis by sex, they found that the estimates for both essential microelements and PUFAs were similar in women and men. This suggests that sex differences did not have a significant impact on the causal relationships between dietary supplementary intake and the risk of ARDs. For example, Fang et al. (92) examined the relationship between the intake of essential micronutrients, including Mg (magnesium), Cu (copper), and K (potassium), and Fe (iron), and the risk of rheumatoid arthritis (RA) among US adults. After adjustment for age, sex, race, BMI, educational level, smoking history, alcohol consumption, family poverty income ratio (PIR), diabetes status and total daily energy intake, logistic regression models and smooth curve fitting were applied to examine the associations of Mg, Cu and K intake with RA.

According to our MR analysis findings, higher levels of EPA have been found to be causally linked to a reduced risk of PsA. Notably, in the FinnGen biobank, female PsA patients make up 55% of the total patient population, while male PsA patients make up 45%. The sex distribution among PsA patients is fairly consistent, lending credibility to the findings indicating a correlation between elevated EPA levels and a lower incidence of PsA in this MR study. However, further investigations are needed to determine the potential impact of PUFAs on other autoimmune rheumatic diseases through future MR studies focusing on sex-specific analysis.

Despite the limitations mentioned, our study has several notable advantages. Firstly, our study utilizes large publicly available GWAS datasets from the UK Biobank, Juvenile Idiopathic Arthritis Genetics Consortium, and FinnGen Biobank, with measures taken to avoid sample duplication. This reinforces the validity and generalizability of our findings. Additionally, by using the MR study design, we effectively address issues of inconsistent selection bias, dietary recall bias, and reverse causation that commonly affect traditional epidemiological studies (93). Furthermore, in order to strengthen the robustness of the results, we conducted multivariable MR analysis.

## 6 Implication for clinical practice

Current treatment options for ARDs are limited, with nonsteroidal anti-inflammatory drugs (NSAIDs), disease-modifying antirheumatic drugs (DMARDs), and biologic agents being the mainstays of therapy. NSAIDs are commonly used to manage pain and inflammation in ARDs, but they do not alter the long-term progression of the disease. DMARDs, such as methotrexate and sulfasalazine, have been utilized for their disease-modifying effects, but their efficacy is variable, and they may have significant side effects. Biologic agents, such as TNF inhibitors and IL-17 inhibitors, have revolutionized the treatment of ARDs by targeting specific inflammatory pathways; however, not all patients respond to these agents, and many patients experience a loss of response over time.

Given the potential disadvantages of medication use in ARDs, there is increasing interest in exploring alternative therapeutic approaches, such as nutritional therapy. Nutritional interventions, including dietary modifications and supplementation with omega-3 PUFAs, have the potential to complement conventional treatments for patients with ARD. Incorporating nutritional support into clinical practice may help optimize disease management and reduce the reliance on pharmacological interventions.

However, there are also contradictory reports addressing the absence of an effect of omega-3 PUFAs and their subclasses on the risk of PsA. Based on MR findings, our study suggested that supplementation or dietary intake of EPA, rather than total omega-3 or DHA, might be beneficial for preventing the onset of PsA, rather than other types of ARDs. These findings shed light on the potential differential impacts of specific omega-3 PUFAs on PsA risk and highlight the importance of considering individual PUFA components when designing prevention strategies for complex ARDs. Overall, this MR study may provide personalized dietary intervention guidelines for patients with ARDs.

Moreover, tailoring dietary recommendations to an individual’s genetic predisposition can optimize treatment outcomes and enhance quality of life for patients with ARDs. Incorporating personalized dietary interventions into clinical practice involves assessing an individual’s PUFA metabolism genes, such as FADS1 and FADS2, and identifying genetic variations that may impact PUFA metabolism and inflammation pathways (94). By combining genetic information with dietary assessments and biomarker monitoring, clinicians can develop personalized dietary plans that are tailored to each ARD patient’s specific needs and goals.

## 7 Conclusion

In conclusion, our extensive analysis using MR revealed EPA as the primary component within omega-3 polyunsaturated fatty acids (PUFAs) that may have a protective effect specifically on PsA, as opposed to other ARDs, such as JIA, SS, gout, and AS. Limited evidence supports the impact of total omega-3 PUFAs and docosahexaenoic acid (DHA) on PsA risk. Additionally, the FADS2 gene appears to play a significant role in mediating the effects of total omega-3 PUFAs, DHA, EPA, and linoleic acid (LA) on PsA susceptibility. These findings suggest that supplementation with EPA, as opposed to total omega-3 PUFAs, total omega-6 PUFAs, DHA, LA, and arachidonic acid (AA), may be a more effective approach for preventing the development of PsA specifically than supplementation with other ARDs, such as JIA, SS, gout, and AS. Further robust epidemiological studies and clinical trials are necessary to further elucidate the potential beneficial mechanisms of high EPA levels in PsA prevention.

## Data availability statement

The datasets presented in this study can be found in online repositories. The names of the repository/repositories and accession number(s) can be found in the article/Supplementary material.

## Author contributions

XiX: Conceptualization, Data curation, Methodology, Writing – original draft. XuX: Conceptualization, Investigation, Methodology, Software, Writing – original draft. MZ: Formal analysis, Methodology, Software, Writing – original draft. S-YW: Conceptualization, Data curation, Formal analysis, Writing – original draft. MY: Conceptualization, Data curation, Investigation, Methodology, Writing – original draft. Y-HW: Data curation, Formal analysis, Writing – original draft. LL: Data curation, Formal analysis, Writing – original draft. Z-lS: Data curation, Methodology, Writing – original draft. R-YW: Conceptualization, Data curation, Formal analysis, Writing – original draft. L-ZM: Formal analysis, Investigation, Methodology, Supervision, Validation, Visualization, Writing – review & editing.

## Footnotes

^1^ http://www.chargeconsortium.com/main/results

^2^ https://www.ukbiobank.ac.uk/
