# Supplementary figures and images for: Assessment of causal relationships between omega-3 and omega-6 polyunsaturated fatty acids in autoimmune rheumatic diseases: a brief research report from a Mendelian randomization study
Source: Front Nutr. 2024 May 28;11:1356207. doi: 10.3389/fnut.2024.1356207 (PMC11165037; doi:10.3389/fnut.2024.1356207)

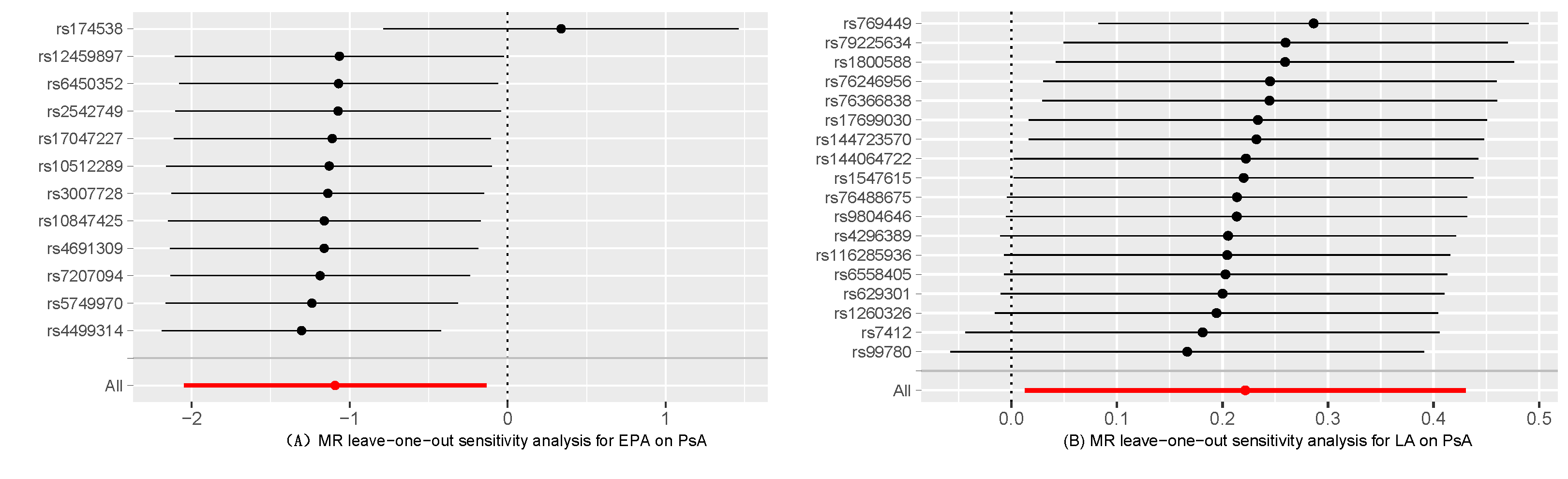

Supplement: Supplementary file 1 [file Data_Sheet_1.ZIP › Supplementary_Fig.1._Leave_one_out.tif]
